# Understanding perspective-taking in multiparty conversations: insights from Mandarin nouns

**DOI:** 10.3389/fpsyg.2025.1499538

**Published:** 2025-01-27

**Authors:** Xiaobei Zheng, Chao Sun

**Affiliations:** ^1^College of International Studies, Shenzhen University, Shenzhen, China; ^2^School of Chinese as a Second Language, Peking University, Beijing, China

**Keywords:** perspective-taking, common ground, Mandarin, bare noun, referential resolution

## Abstract

Individuals frequently adopt others’ perspectives both when interpreting language and when formulating their own responses in conversation. This experiment tested how participants used perspective information to resolve references for bare nouns in Mandarin. Specifically, it explored whether, when faced with two interlocutors, participants distinguished between each individual’s perspective or considered both as a whole. Using a classical referential game, the study manipulated the visual perspectives of two partners. In Experiment 1, both speakers had the same seating direction and visual field, and the results showed that participants equally took their perspectives into account above chance levels, providing a baseline finding for referential resolution of Mandarin bare nouns in perspective-taking studies. In Experiment 2, both speakers had the same seating direction but one of them shared the larger portion of visual field with the participants. The results showed that participants took the perspectives of the two speakers independently, while also comparing the perspectives of both interlocutors to facilitate quicker and more accurate referential resolution. These findings demonstrate that perspective-taking is a complex and dynamic process, providing evidence for the study of perspective-taking in Mandarin and contributing insights into comprehension processing in multiparty conversations.

## 1 Introduction

### 1.1 Common ground in communication

During daily communication, in addition to employing linguistic knowledge to construct discourse, interlocutors consider the physical background, the information known to other parties, and other contextual factors. One fundamental factor is the common ground shared between communication participants (Stalnaker, 1978). Common ground refers to the knowledge, beliefs, and information mutually understood by interlocutors. In language philosophy, it includes a deeper dimension, such as the awareness interlocutors have of their partners’ awareness of this shared knowledge, beliefs, and information (Schiffer, 1972; Clark and Marshall, 1978; Aumann, 1976). In psycholinguistics, researchers mainly investigate how shared information affects online language production and comprehension, a process referred to as perspective-taking during conversation.

Psycholinguistic studies often explore how individuals use both privileged and common ground information during referential processing. Privileged ground refers to knowledge known only to one interlocutor, while common ground consists of information shared by both. Research shows that although common ground facilitates faster and more accurate comprehension, participants occasionally consider their privileged ground (Heller and Brown-Schmidt, 2023; Heller et al., 2016; Mozuraitis et al., 2018; Ryskin et al., 2020; Keysar et al., 2000; Chambers et al., 2008; Epley et al., 2004), particularly when interpreting questions posed by others (Brown-Schmidt et al., 2008).

### 1.2 Strategies in perspective-taking

To resolve the debate regarding the use of common vs. privileged ground in conversation, psycholinguistic studies have focused on the timing of processing. Some studies propose an initial egocentric strategy, where people prioritize their own knowledge and attentional states and then adjust based on common ground (Keysar et al., 2000; Keysar et al., 1998). Other research suggests that individuals begin with an initial consideration of common ground, treating it as one factor in top-down language processing that operates alongside other linguistic factors (Hanna et al., 2003; Heller et al., 2012; Nadig and Sedivy, 2002; Sikos et al., 2019; Rubin et al., 2011).

Evidence supporting both proposals has accumulated, but results are often influenced by experimental design. Consequently, some theories propose that perspective-taking does not follow an “all-or-none” approach. Certain models blur the distinction between privileged and common ground. For example, the memory-based model suggests that ground-related information is stored as a general episodic memory, which is later retrieved through resonance with relevant cues during language processing (Horton and Gerrig, 2005a, 2005b, 2016). If, for instance, Tom received a phone call from his boss while Mary was present, Mary would serve as a cue for Tom to retrieve the memory of the call later, even if the content of the call did not relate to their common ground. Similarly, when information lacks strong cues, individuals may rely on an egocentric strategy, even if that information exists in the common ground. This proposal helps resolve previous debates on the use of egocentric strategies versus common ground.

Another proposal, partner-specific processing, suggests that individuals establish different common grounds with different individuals. This proposal is also integrated with the memory-based model by emphasizing the unique role of humans. That is, among all kinds of memory cues, humans—as social agents—are the most powerful triggers, making information jointly attended to by both partners more easily retrievable for later use (Brown-Schmidt, 2012; Brown-Schmidt et al., 2015; Brown-Schmidt and Hanna, 2011). In the phone call example, if Tom had been watching TV when he answered the call, the TV show might act as a cue for later retrieval. However, compared to the TV show, Mary would be a stronger cue when recalling the content of the call.

Research on conceptual pacts supports this partner-specific explanation. Specifically, individuals tend to maintain the same expressions when conversing with the same partner but abandon them when interacting with a new partner (Metzing and Brennan, 2003; Brown-Schmidt, 2009; Kronmüller and Barr, 2007). Similar proposals have also been made in conversational theories. For instance, Pickering and Garrod (2004, 2013, 2021) emphasized cognitive alignment, where participants unconsciously align their language at various levels, such as syntax and vocabulary, to facilitate communication. The process of cognitive alignment also involves memory, integrating previous linguistic information with the partner’s identity.

Philosophical discussions of common ground also address the influence of interlocutors-related information and the processing mechanisms associated with it. From a philosophical perspective, common ground entails an infinite regress, where each participant infers that their partner knows that they know a given proposition, continuing indefinitely. Moreover, common ground is seen as a dynamic entity that evolves throughout communication. Therefore, such processing places considerable cognitive demands on interlocutors. To address this challenge, Clark and Marshall introduced the concept of the co-presence heuristic, where common ground is established based on a shared physical environment, prior discourse, and cultural background (Clark, 1996; Clark and Marshall, 1981). Using these heuristics, speakers do not need to assess whether every piece of information is in or out of the common ground. For example, when discussing the only visible book between interlocutors, one might use minimal description, such as “the book” or even just “it.” The physical co-presence narrows down the referential domain without requiring deeper assessment. This explanation parallels the concept of joint attention in developmental psychology, which describes how infants and adults coordinate their focus on the same object, facilitating accurate reference resolution (Tomasello and Farrar, 1986). In both cases, physical co-presence contributes to establishing common ground between interlocutors.

These heuristics also take cultural and social background factors into account. For instance, when a doctor explains an illness to a patient, they may avoid using medical jargon, but might use more technical language when speaking to a colleague. This social and cultural background can be seen as a holistic strategy in which individuals adopt others’ perspectives based on their social identity or stance, allowing for quicker evaluations. The assessment of overall similarity facilitates communication by promoting mutual understanding and reducing misinterpretations (Runkel, 1956). These philosophical and empirical discussions show that people integrate information about the communicative partner’s identity, social background, and the surrounding context to form certain expectations and strategies that help them communicate more effectively.

### 1.3 Research questions in partner-specific processing

In summary, the aforementioned explanations suggest a dynamic, non-binary approach to processing perspective in language comprehension. Whether focusing on partner-specific or cultural heuristics, these studies emphasize the importance of interlocutor-related information. However, the interlocutor-related information is quite broad.

First, interlocutor-related information has different categories at the social and cognitive levels. In cultural heuristics, the partner is seen as part of a social group with a certain background. For example, the conversational partner could be a patient of the doctor or a colleague of the doctor. However, in memory-based and cognitive alignment models, the information about the interlocutor shifts to memory or perceptual priming, which is an automatic process. For instance, when conversation partners sit face-to-face, factors such as the direction of their seats or their line of sight might involve only the automatic processing of the physical environment.

Second, how the partner-related information or similarity between the partner and oneself influences perspective-taking remains unclear. It may lead to either an egocentric or collective strategy. Studies suggest that people overestimate the mental states of familiar individuals and display more egocentrism towards the perspectives of friends compared to strangers (Savitsky et al., 2011; Robbins and Krueger, 2005). The similarity at the cognitive level has also been examined experimentally. High information overlap between communicators can reduce communication effectiveness, as speakers tend to overestimate listeners’ knowledge (Wu and Keysar, 2007a). As shared information increases, people are more likely to overestimate common ground (Zheng and Breheny, 2021). Research on child development provides similar evidence (Moll and Meltzoff, 2011; Moll et al., 2007, 2011). In these studies, two adult speakers engage with a child, each establishing distinct common ground through joint attention. The findings suggest that children under the age of two can differentiate between the common ground they share with different partners. However, in some cases, even when adults do not share information with children, children may still treat the adult as a knowledgeable party (Moll et al., 2011).

Last but not least, this study focuses on two additional aspects. First, it expands the scope of conversational analysis by moving beyond traditional dyadic interactions to triadic conversations. This shift introduces more complex scenarios, as the knowledge systems of the three participants involve overlapping layers of common ground between each pair. Second, the study emphasizes the comprehension processes of the addressee, rather than the production processes of the speaker. Specifically, it examines how the addressee interprets discourse based on the perspectives of others, as opposed to how speakers plan their discourse from their audience’s perspective.

Based on this framework, we address two key research questions: (1) Does the addressee distinguish between the perspectives of the two speakers, leading to distinct understandings of their utterances? (2) Does the common ground shared between the two speakers and that between the addressee and each speaker interact in ways that shape the addressee’s comprehension strategies? We hypothesize that addressees will adopt the perspectives of each speaker based on their respective visual fields; however, increased similarity between speakers and addressees may either enhance interpretive accuracy or lead to overly egocentric understandings. This study aims to provide empirical evidence to clarify these possibilities.

### 1.4 The present study

The present experiment employed a classic referential game in which speakers instructed participants to manipulate target objects on a shelf (Keysar et al., 2000). Participants and speakers sat on opposite sides of the shelf, which contained both transparent and opaque compartments, resulting in different sets of object information visible to the participants and the speakers. This setup allowed us to explore how participants adjust their interpretation of the speakers’ instructions based on the varying levels of shared information.

Each participant engaged in the game with two confederate speakers, who alternated giving instructions. The purpose of this was to compare how participants analyse and integrate each speaker’s perspective in the same communicative context. On the one hand, both confederates acted as speakers, seated opposite the participant, identical in their role and physical positioning, but distinct from the participant. This setup ensures that both speakers in the task have exactly the same role, i.e., as directors, and are seated in the same position, directly facing the participants. On the other hand, the two confederates may or may not share the same information with the participant. This setup allows for the manipulation of the common ground between the participants and each speaker. The study aims to investigate how the different levels of common ground influence participants’ perspective-taking and language comprehension. We predict that participants will interpret different speakers’ discourse based on the specific shared knowledge associated with each speaker, consistent with findings from previous studies on perspective-taking. Moreover, participants are also expected to partially integrate the perspectives of the two speakers because they share a common role in the discourse.

Additionally, the instructions were delivered in Mandarin, where nouns can stand alone without a determiner, a construction known as bare nouns. For example, the phrase “pick up dog” in Mandarin does not require a determiner. Bare nouns in Mandarin can convey definiteness, indefiniteness, or generality depending on the context (Li and Thompson, 1975, 1989). In previous referential studies conducted in English, the inclusion of the determiner “the” before a noun, as in “pick up the dog,” typically leads the addressee to interpret the noun as definite, thereby encouraging perspective-taking. However, in Mandarin, bare nouns do not inherently imply such a preference. As a result, “pick up dog” can be interpreted as referring to a specific, indefinite, or generic dog. When multiple dogs are present, participants may reasonably consider picking up any, some, or all of them. This lack of grammatical constraint provides an opportunity to observe how participants make their choices in a more flexible linguistic environment.

In Experiment 1, since research on perspective-taking in Mandarin is relatively limited, we first conducted an exploratory experiment in which both confederates had identical identities and shared knowledge. We expected that participants will show no difference in their interpretation of the two confederates’ instructions. Additionally, Experiment 1 will provide a baseline for participants’ level of perspective-taking in this experimental design.

## 2 Experiment 1

### 2.1 Participants

Twenty-seven university students (14 female and 13 male, aged 17 to 24 years) participated in the experiment. Two female research assistants who were trained as confederates also took part in the experiment. All participants were recruited through campus advertisements and were informed that they would receive a cash reward regardless of their performance. They were also told they had the right to withdraw from the experiment at any time and still receive the reward. All participants are native Mandarin speakers and have lived in Mandarin-speaking communities since childhood. They were university students with similar levels of proficiency in Mandarin. None of the participants reported any cognitive, hearing, vision, or other relevant impairments.

### 2.2 Methods

#### 2.2.1 Stimuli and design

**Figure 1:**
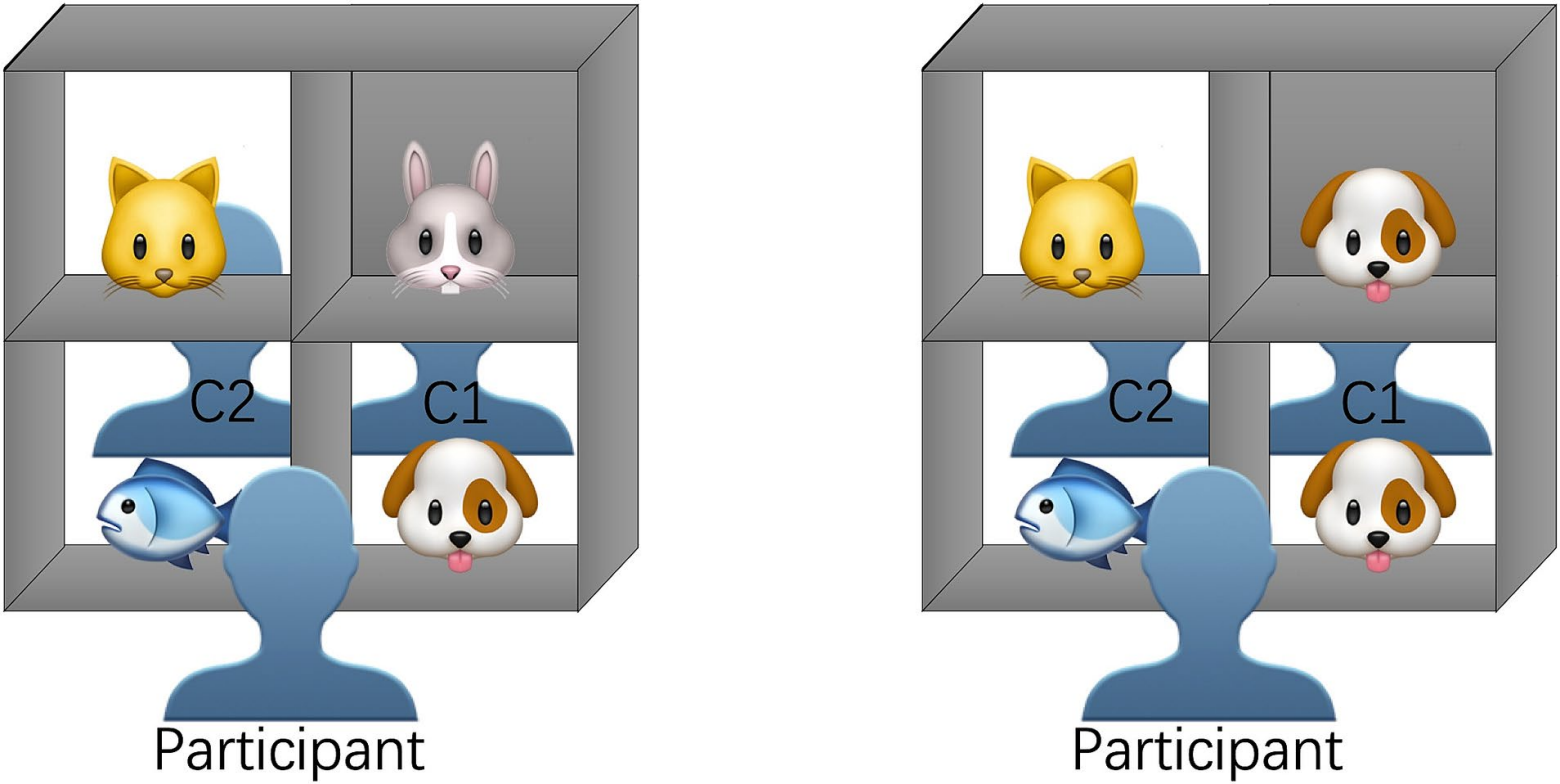
The display of the shelf and the seating arrangement of participants and confederates in Experiment 1.

The experiment was conducted in a quiet laboratory with a table placed in the center of the room. A shelf was positioned on the table. During the experiment, two cameras were used to record the participants’ behavior and eye movements. The camera recording behavior was placed behind the laboratory curtains, while the camera recording eye movement trajectories was set up behind the shelf. In the experiment, participants sat at the front of the shelf, and two confederates were seated on the opposite side of the shelf. The shelf contained four compartments. Three compartments were transparent, making the objects in these compartments visible to both the participants and the two confederate speakers. These objects were shared objects. One compartment, however, was blocked by a partition, so the objects inside were visible only to the participants seated at the front. This object was referred to as an unshared object (as illustrated in Figure 1).

During the experiment, in each trial, the participants saw pictures of four objects placed in the four compartments. These objects included items such as animals, fruits, and vehicles. In each trial, the objects belonged to the same category; for instance, all animals. The arrangement of the cards in the compartments was manipulated according to competitor-type conditions. In the non-competitor condition, the four objects were different; for instance, a cat, a fish, a rabbit and a dog (Figure 1 left). In the competitor condition, the blocked compartment contained an object identical to one in a transparent compartment; for instance, a cat, a fish and two dogs (Figure 1 right). For comparative analysis, the non-competitor and competitor conditions were paired, meaning that the three shared objects in the transparent compartments and their positions were identical across conditions, with the only difference being whether the unshared object in the blocked compartment was a duplicate.

In each trial, the confederates’ instructions were to “please pick… (the object name).” In the competitor condition, the critical object referred to the one present in both the blocked and transparent compartments. For participants seated in front of the shelf, this instruction was semantically ambiguous because it could refer to either the shared or the unshared object. In the non-competitor condition, the confederates used the same name, referring to the shared object in the transparent compartment without ambiguity. For example, in the sample display in Figure 1, the critical noun “dog” in the instruction “please pick (the) dog” could refer to either the shared or unshared dog in the competitor condition, but there was only one dog, which is shared, in the non-competitor condition. The participant and the two confederates could see each other, but to avoid the confederates’ attention to objects giving hints to the participant, the confederates avoided making eye contact with the participant during the critical conversation.

To prevent participants from developing strategies during the experiment, such as immediately selecting the object in the transparent compartment when there was an identical object competitor in the blocked compartment, a filler condition was introduced. In this condition, the filler trials randomly used the same objects and arrangement as the experimental condition, but with two identical objects included. Unlike in the competitor condition, the two identical objects were not necessarily placed in the blocked compartment. Importantly, the confederates’ instructions did not refer to these duplicate objects.

Moreover, the identity of the speakers constituted an additional experimental condition. Half of the trials were conducted under the instruction of Confederate 1 (C1), while the remaining half were under the guidance of Confederate 2 (C2), with both individuals providing identical instructions. These instructions were presented in a randomized sequence, with adjustments made for cases where two identical instructions from different speakers appeared within a span of three consecutive trials.

In summary, each participant received 48 trials. The competitor types served as a within-participant variable, with participants receiving 16 non-competitor trials and 16-competitor trials. An additional 16 filler trials were included. The confederate identity served as another within-participant variable, with each confederate providing 24 instructions.

#### 2.2.2 Procedure

Upon arrival at the laboratory, participants were informed that they would engage in a cooperative task. They would be assigned as “operators,” who were instructed to examine objects placed on a shelf by two other “instructors” (Confederate 1 and 2). The participants were told that the two confederates had previously functioned as operators and were now tasked with acting as instructors in the present task. To ensure the credibility of their conversations during the repeated picking task, the participants were also told that the two confederates were performing an additional task requiring the participants’ assistance.

Next, to help participants familiarize themselves with the confederate’s view, they were asked to sit on the opposite side of the shelf and complete several practice trials as instructors. Additionally, when the confederate acted as the operator, regardless of how many objects were visible to her, she would select only one object. This design aimed to convince participants that the confederates’ extra task required just one object. In the formal test, no participant chose two or more objects.

Once the formal experiment began, confederates alternated in providing instructions. During the initial trials, they might feign hesitation, pretending that they were considering their own extra task. To minimize potential delays in participants’ eye movements and behavioral data resulting from unfamiliarity during the initial phase, the first five trials of the experiment were all filler trials. This enabled participants to acquaint themselves with the task before engaging with the critical trials.

#### 2.2.3 Coding

For the behavioral data, the experimenter recorded the objects chosen by the participants during the experiment. After the experiment, another research assistant re-coded the participant’s choices based on the video recording. Finally, a third research assistant verified the records from the two coders and resolved any inconsistency using the video recordings. The participants’ selection of target items was calculated. “Target items” refer to the object mentioned by the speaker in the non-competitor condition, as well as the object in the transparent grid mentioned by the speaker in the competitor condition. Each participant completed 8 trials for each condition, so the target choice proportion for each participant was calculated by dividing the number of target choices by 8, yielding four percentage scores for each participant under the four combinations of speaker and competitor types.

For the eye movement data, two trained research assistants coded the eye movement data based on the video recordings, with the coders blind to each trial’s condition and the specific object arrangement. The coders performed frame-by-frame coding of the participant’s gaze direction. The eye movement data were recorded at a frequency of 25 frames per second, which corresponded to fixation intervals of 40ms. Eye-tracking data concerning the target object were analyzed across the interval corresponding to the duration of the critical noun (e.g., “dog”), specifically from 200ms to 1200ms after word onset. The time window began at the 200ms mark following the onset of the critical word, in line with the standard assumption regarding the time necessary to program and execute an eye movement (Hallett, 1986). The time window ended at 1200ms due to the average latency at which participants began to select the objects. The coding followed the same method as Huang et al. (2013), with the fixation quadrant indicating the category of the object, which was further divided into target and non-target objects. The average proportion of looks to the target in each time bin was calculated by dividing target fixations by all fixations across the four objects.

### 2.3 Results

#### 2.3.1 Behavioral data analyses

**Figure 2:**
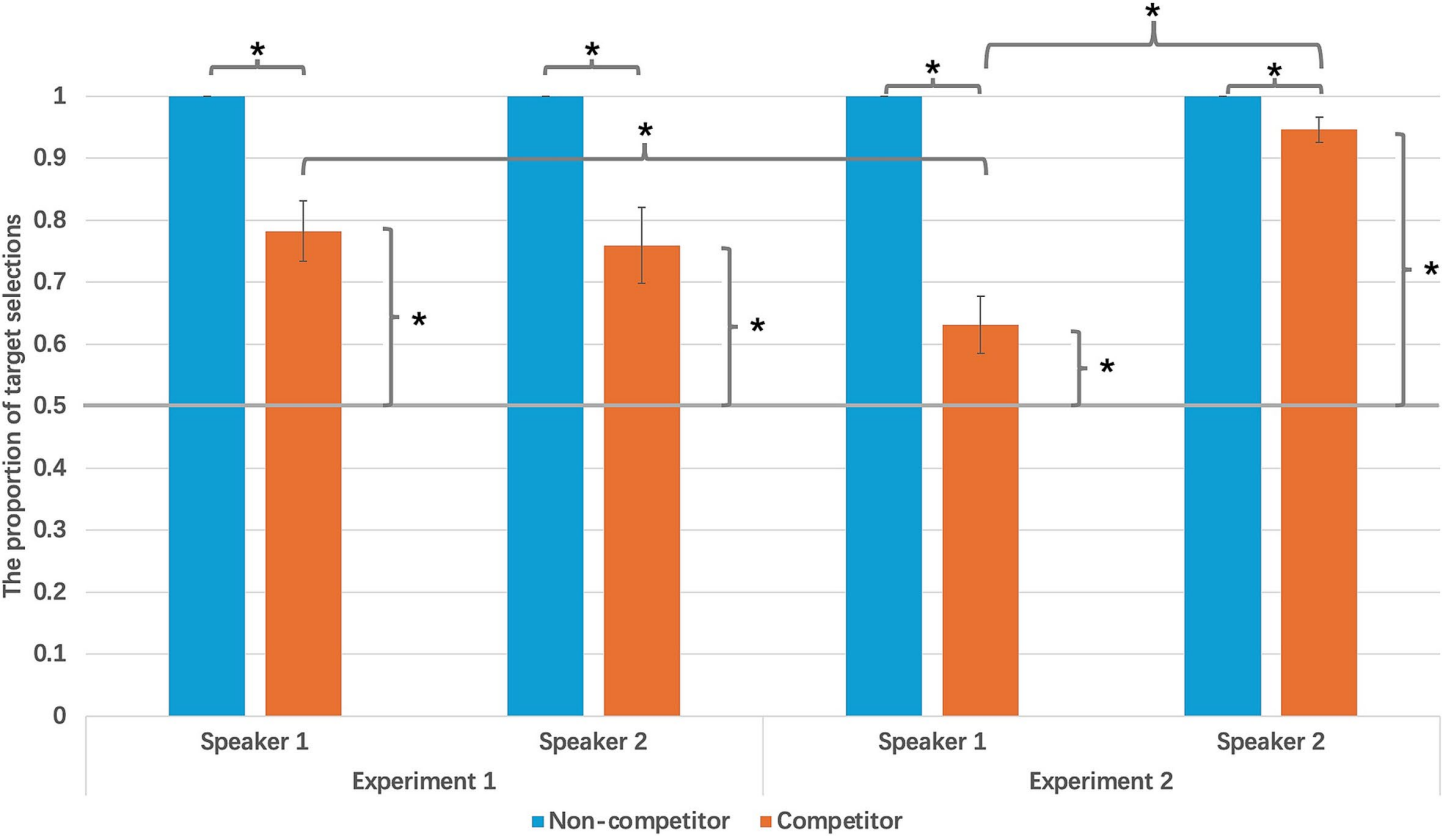
Proportion of participants’ selection of the target object from two confederates across two competitor types in Experiments 1 and 2.

First, Wilcoxon signed ranks tests were conducted to examine the effect of competitor types on target choice, with Confederate 1 and 2 trials analysed separately. Therefore, competitor type (competitor vs. non-competitor) served as the independent variable, and the proportion of target selection served as the dependent variable (Figure 2).

In C1 (Confederate 1) trials, results showed a significant difference between competitor and non-competitor trials, Z = −3.422, *p* = 0.001, effect size = 0.88. Participants selected the target item more frequently in non-competitor trials (M = 1, SD = 0) compared to competitor trials (M = 0.78, SD = 0.25). Similarly, in C2 (Confederate 2) trials, a significant effect of competitor type was also observed, Z = −3.179, *p* = 0.001, effect size = 0.75. As in condition C1, participants were more likely to select the target item in non-competitor trials (M = 1, SD = 0) than in competitor trials (M = 0.76, SD = 0.32). These findings indicate that the presence of a competitor item reduced the likelihood of participants selecting the target item.

Secondly, the effects of speaker identity in competitor trials was explored by a Wilcoxon signed ranks test. The results showed that no significant difference between the target choice proportions in C1and C2 during competitor trials, Z = −0.752, *p* = 0.452, effect size = 0.069. This result indicates that the presence of a competitor similarly impacted participants’ target selection across both speakers, with no observable variation between the two.

Thirdly, participants’ target choice in the competitor condition was compared to the chance level of 0.5. Two separate one sample Wilcoxon signed ranks tests were performed for C1 and C2 trials. Results showed that for both C1 and C2, participants’ target choice was significantly higher than chance, C1: Z = 3.893, *p* < 0.001, effect size = 1.12; C2: Z = 3.527, *p* < 0.001, effect size = 0.813, suggesting that participants were able to reliably select the target item despite the presence of a competitor item, significantly exceeding chance-level performance.

The results suggest that participants utilize perspective information to modulate the referential domain of target nouns to some extent. The word labels for targets were bare nouns. In Mandarin, bare nouns can refer to definite, indefinite or generic candidates, which implies a broader referential domain. However, participants’ referential resolution to the shared items is above chance, showing that they used perspective cues to solve the referential resolution.

#### 2.3.2 Eye-tracking data analyses

**Figure 3:**
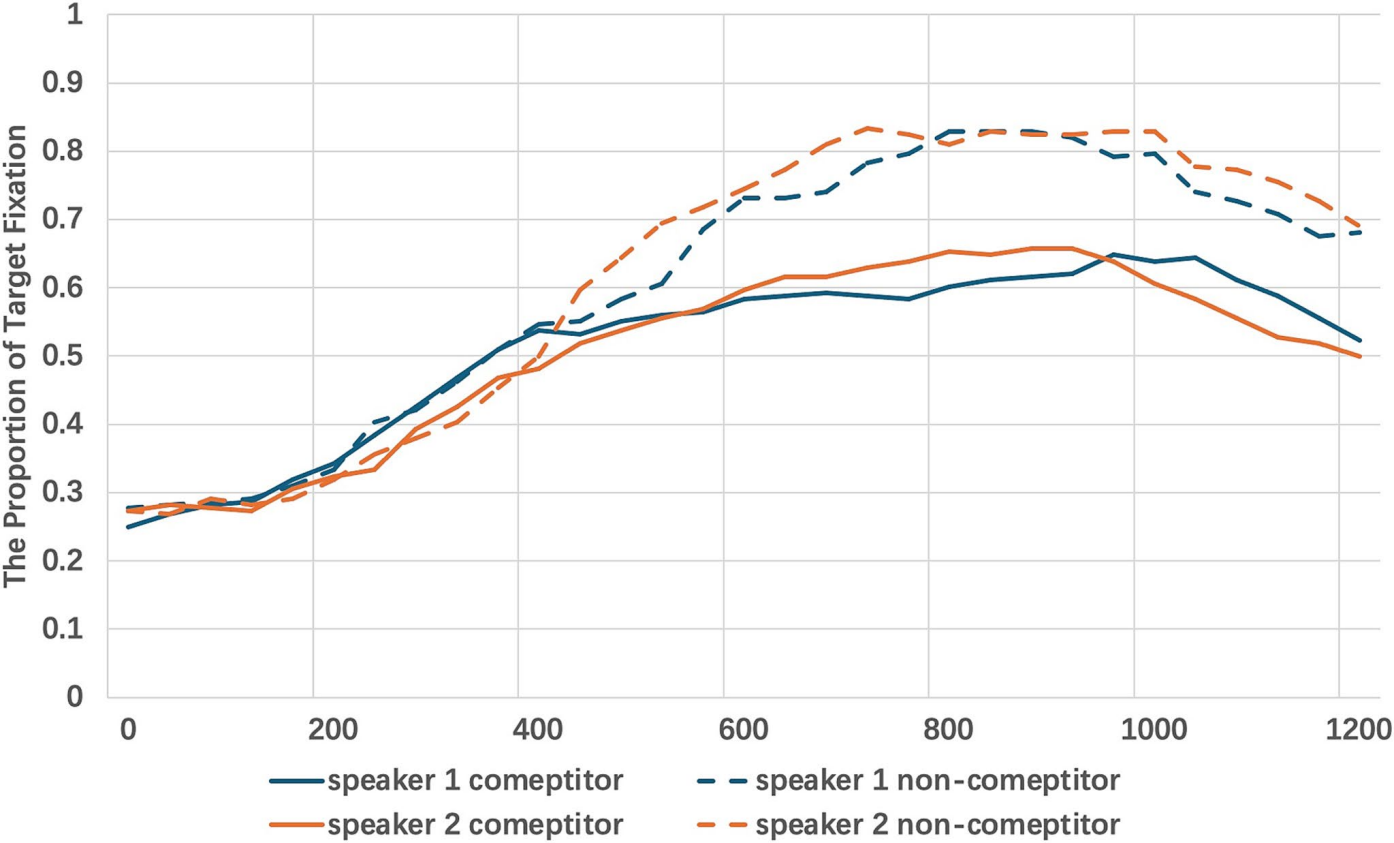
The 1200ms time window capturing participants’ eye movements from the onset of the target object description across speakers and competitor types.

A general linear mixed-effects model was conducted using the lmer function in R to examine the impact of competitor type and speaker on participants’ eye movements. The model included competitor type (competitor vs. non-competitor) and speaker (C1 vs. C2) as fixed effects, while participants and items were entered as random effects. The dependent variable was the proportion of target fixation. The fixation towards the target was graphed over time in Figure 3.

The results indicated a significant main effect of competitor type *β* = 0.10, SE = 0.03, t = 2.57, *p* < 0.05, suggesting that participants had significantly higher fixations to the target in the non-competitor condition compared to the competitor condition. The effect of speaker was not significant, β = 0.01, SE = 0.01, t = 0.19, *p* = 0.84, indicating no substantial difference in target fixations based on speaker identity. The interaction between speaker and competitor type was also not significant, β = 0.01, SE = 0.01, t = 0.71, *p* = 0.47, suggesting that the effect of competitor type on target fixation did not vary significantly across different speakers. Overall, the results exhibited patterns consistent with the behavioral data, highlighting a significant impact of competitor type on eye movements, while speaker identity and its interaction with competitor type did not significantly influence fixation patterns.

### 2.4 Discussion

In this experiment, participants collaborated with two confederates on an object-picking task. While all four candidate objects were visible to the participants, only three were visible to the confederate speakers. The results of this study indicate that, compared to the non-competitor condition, participants were influenced by their privileged knowledge and considered the unshared competitor objects more frequently. However, compared to the chance level, participants still tended to adopt the confederates’ perspective and selected the shared target objects.

The results are consistent with predictions. Rationally, when the confederates referred to an object that had a duplicate competitor in the participants’ privileged view, participants were expected to consider the confederates’ perspective and exclude the competitor object from the referential domain. However, previous studies have also found that participants consider their privileged competitor object to some extent, given that the competitor is semantically consistent with the word label (Keysar et al., 2000). The effect of competitor types observed in this study supports this conclusion.

Given that few studies within this field of research have been entirely conducted in a Mandarin-speaking context, the present study provides exploratory results that could serve as a baseline for future studies. On the one hand, cross-cultural research suggests that in collectivist cultures like Chinese culture, people are more inclined to consider others’ perspectives and focus more on the information in the common ground (Wu and Keysar, 2007b). On the other hand, Mandarin allows for bare nouns, which permit generic or category-level reference. Previous studies have shown that Mandarin bare nouns exhibit flexibility in interpretation, depending heavily on semantic and pragmatic cues in the discourse context (Kuo, 2008; Rullmann and You, 2006; Chang, 2016). That is, even if participants selected the unshared competitor objects, this selection could be considered acceptable. Unlike in English, where the determiner “the” in noun phrases provides additional cues for definite reference in common ground, bare nouns in Mandarin offer fewer such cues. Nevertheless, the results in this study are generally consistent with previous findings from English-language contexts, as participants’ selection of the shared object was above chance level, demonstrating participants’ perspective-taking process.

The next experiment will explore the main question of interest in this research: In perspective-taking, which is more significant: one’s general stance or the specific shared information? In the following experiment, all experimental settings will remain the same, except that Confederate 1 will be able to see the objects in the blocked compartment through a gap between the partition. This means that Confederate 1 shares the same information with the participant, but they still share the same stance with Confederate 2 by seating together as speakers to guide the participant in completing the task. Should the results of the following experiment show the same trend as those of the first, exhibiting a similar gaze pattern during the competitor condition for both Confederate 1 and 2 trials, it would suggest that the general stance of the confederates may be influential. Conversely, if the results diverge from those of the first experiment, particularly with distinct processing of Confederate 1 and 2 trials, it would indicate a more significant role for shared knowledge inference. Furthermore, the shift in gaze pattern in Confederate 1 and 2 trials will also be valuable for further discussion, as it may offer deeper insights into the change in perspective-taking strategy when the similarity among the speakers and addressee changes.

## 3 Experiment 2

### 3.1 Participants

Twenty-one participants took part in Experiment 2 (11 female and 10 male, aged 17 to 23 years). The same two confederate speakers participated as in the first experiment. The recruitment and rewarding procedures were identical to those in Experiment 1. All participants are native Mandarin speakers, and none of the participants reported any cognitive, hearing, vision, or other related issues.

### 3.2 Method*s*

**Figure 4:**
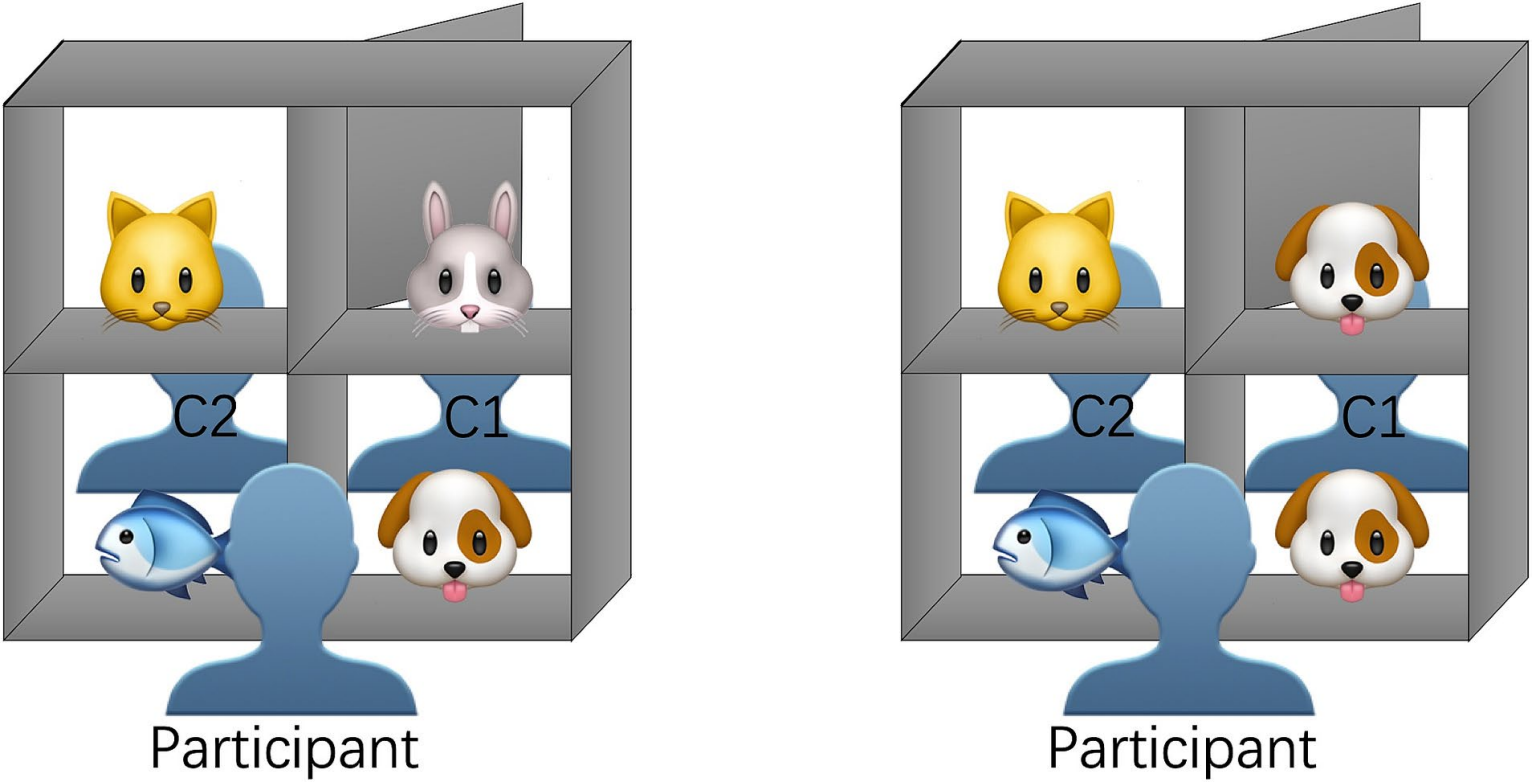
The display of the shelf and the seating arrangement of participants and confederates of Experiment 2.

Experiment 2 used the same materials and procedure as Experiment 1. The only difference was that Confederate 1 could see the objects in the blocked compartment (see Figure 4). During the test, participants were first instructed to familiarise themselves with the shelf from the opposite side by sitting in the positions of C1 and C2 for several practice trials. In the formal test, the two confederate speakers took turns asking referential questions to the participants.

This experiment also consisted of 48 trials, including 16 non-competitor trials, 16 competitor trials, and 16 filler trials. Trials were evenly divided between C1 and C2, with each having 24 trials. The order of instructions was identical to that in Experiment 1, ensuring the two sets of experiments were comparable.

### 3.3 Results

#### 3.3.1 Behavioral data analyses

In the second experiment, similar analyses were conducted to examine the effects of competitor types on target selection. As with the first experiment, Wilcoxon signed ranks tests were conducted separately for the trials in conditions C1 and C2, with competitor types (competitor vs. non-competitor) as the independent variable and target selection proportion as the dependent variable (Figure 2).

The results mirrored those of the first experiment. In C1 trials, participants selected the target item more frequently in non-competitor trials (M = 1, SD = 0) than in competitor trials (M = 0.63, SD = 0.21), Z = −4.03, *p* < 0.001, effect size = 1.762. Similarly, in condition C2, participants also selected the target item more often in non-competitor trials (M = 1, SD = 0) than in competitor trials (M = 0.95, SD = 0.09), Z = −2.53, *p* < 0.05, effect size = 0.556. This confirms that the presence of a competitor item significantly reduced participants’ likelihood of selecting the target item, consistent with the results from the first experiment.

However, unlike in the first experiment, a paired samples Wilcoxon signed ranks test for the competitor trials revealed a significant difference between C1 and C2. Participants’ target selection proportions in C1 were significantly lower than in C2, Z = −3.94, *p* < 0.001, effect size = 1.981. This suggests that whether the particular information is shared had a significant impact on how participants resolve the referential ambiguity.

Participants’ target choice in the competitor condition was also compared to the chance level of 0.5. Two separate one-sample Wilcoxon signed ranks tests were performed for C1 and C2 trials. Results showed that for both C1 and C2, participants’ target choice was significantly higher than chance, C1: Z = 2.47, *p* < 0.05, effect size = 0.619; C2: Z = 4.17, *p* < 0.001, effect size = 5, suggesting that participants’ target choices in both C1 and C2 conditions in the competitor trials were significantly greater than the chance level of 0.5.

The results, on the one hand, suggest that participants refer to the shared information to constrain the referential domain, given the effect of speakers in the competitor trials. On the other hand, it also suggests that the general stance of the speakers influences how participants interpret the bare noun, given that even in C1 trials they chose the C2 shared target above chance level.

#### 3.3.2 Combined analysis of Experiments 1 and 2

To further explore the effects of shared knowledge across both experiments, a combined analysis was conducted, focusing on the competitor conditions in C1 and C2 trials separately.

For C1, a Mann–Whitney U test revealed a significant difference between the competitor trials in Experiments 1 and 2, Z = −2.440, *p* < 0.05, effect size = 0.611. Participants selected the target item less frequently in Experiment 2, indicating a stronger effect of the competitor item when the confederate shared both referential candidates with the participants. For C2, no difference was found between Experiments 1 and 2 in competitor trials, Z = −1.621, *p* = 0.105, effect size = 0.727. This result is consistent with expectations, as C2’s visual field remained unchanged, while in C1, both referential candidates were visible in Experiment 2. Since C2’s position and visual field remained the same across both experiments, no difference across C2 was predicted. In the next section, participants’ eye movements will be analysed to further explore their potential processing strategies.

#### 3.3.3 Eye-tracking data analyses

**Figure 5:**
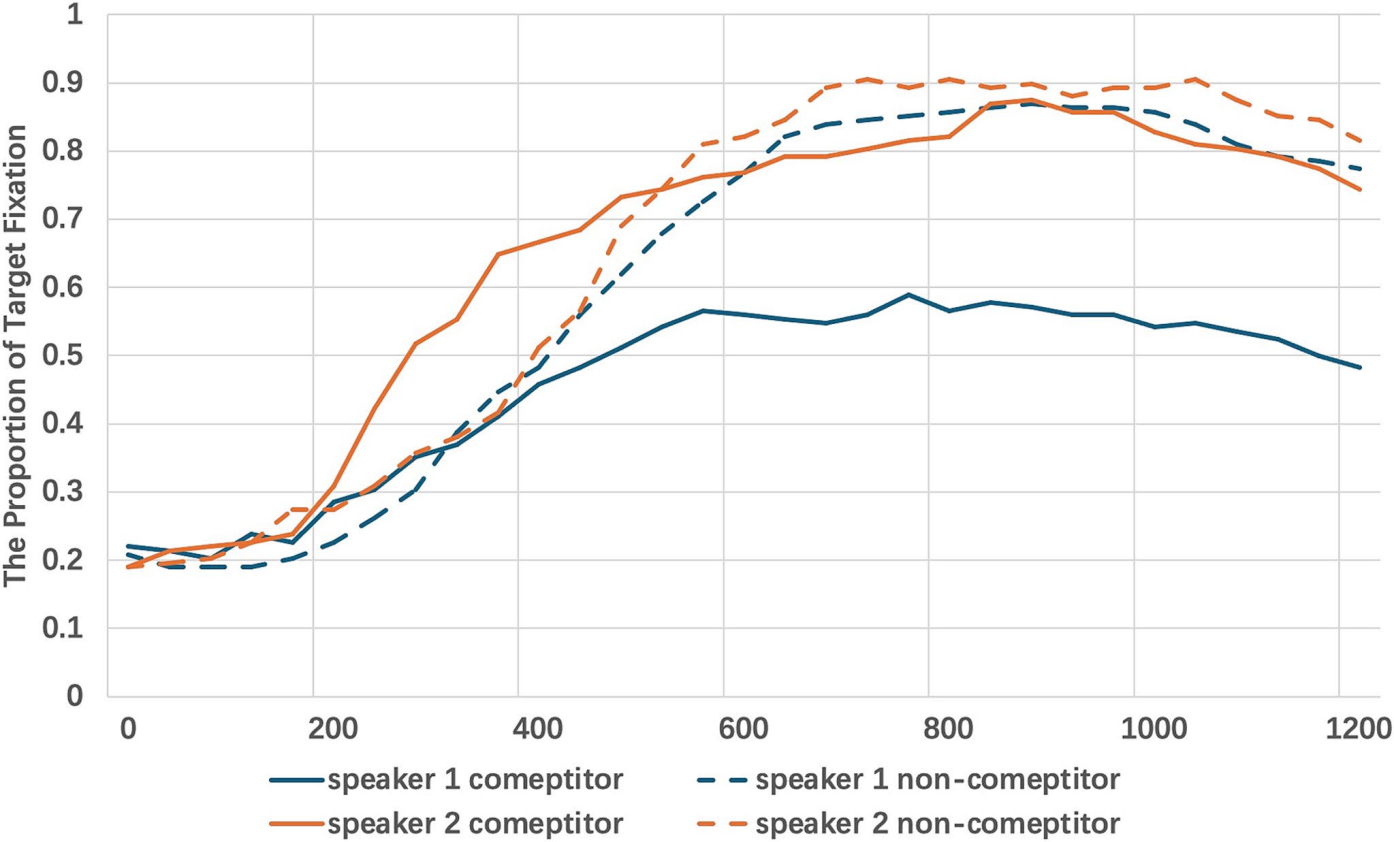
The 1200 ms time window capturing participants’ eye movements from the onset of the target object description across different conditions and speakers.

As the first experiment, a general linear mixed-effects model was conducted using the lmer function in R to examine the impact of competitor type and speaker on participants’ eye movements. The model included competitor types (competitor vs. non-competitor) and speaker (C1 vs. C2) as fixed effects, while participants and items were incorporated as random effects. The dependent variable was the fixation to target, measured in 40ms bins. The fixation towards the target was graphed over time in Figure 5.

The analysis revealed a significant main effect of competitor type, *β* = 0.37, SE = 0.05, t = 7.21, *p* < 0.001, indicating that fixations to the target were significantly higher in non-competitor trials compared to competitor trials. Additionally, a significant effect of speaker was observed, β = 0.23, SE = 0.03, t = 8.03, *p* < 0.001, suggesting that fixations to the target varied depending on the speaker’s identity. Furthermore, a significant interaction between competitor type and speaker was found, β = −0.19, SE = 0.02, t = −12.764, *p* < 0.001, demonstrating that the effect of competitor type on target fixation differed across speakers.

To further explore the interaction between competitor type and speaker, post-hoc analyses were conducted using the emmeans package in R. The purpose was to clarify how the effect of competitor type on fixation to the target differed across speakers, as well as how the speaker effect varied between competitor and non-competitor conditions.

The analysis revealed significant differences between speakers in the competitor condition, estimate = −0.23, SE = 0.03, z = −8.04, *p* < 0.001, indicating that fixations to the target were significantly lower for C1 compared to C2 when competitors were present. In contrast, the comparison between speakers in the non-competitor condition was not significant, estimate = −0.04, SE = 0.03, z = −1.29, *p* = 0.20, suggesting that there were no notable differences in target fixations between C1 and C2 when no competitors were present.

The effect of competitor type was also analysed separately for each speaker. For C1, fixations to the target were significantly higher in the non-competitor condition compared to the competitor condition, estimate = −0.18, SE = 0.05, z = −3.81, *p* < 0.001. However, for C2, this effect was not significant, estimate = 0.02, SE = 0.05, z = 0.32, *p* = 0.75, indicating no substantial difference in target fixations between competitor and non-competitor conditions.

#### 3.3.4 Combined analysis of Experiments 1 and 2

A general linear mixed-effects model was conducted to investigate the effects of competitor type (competitor vs. non-competitor) and experiment (experiment 1 vs. experiment 2) on participants’ eye movements, with separate analyses for each speaker. The model included competitor type and experiment as fixed effects, and participants and items as random effects. The dependent variable was fixation to the target, measured in 40 ms bins.

For C1, the model revealed significant effects of both experiment and competitor type. The effect of experiment was significant, *β* = 0.05, SE = 0.01, t = 5.28, *p* < 0.001, indicating that fixation patterns differed between experiment 1 and experiment 2. The effect of competitor type was also significant, β = 0.24, SE = 0.05, t = 4.37, *p* < 0.001, with greater fixations to the target in the non-competitor condition compared to the competitor condition. Moreover, the interaction between experiment and competitor type was significant, β = −0.06, SE = 0.01, t = −4.11, *p* < 0.001, suggesting that the effect of competitor type on target fixation varied across experiments.

Post-hoc contrasts indicated a significant difference in fixation to the target between experiment 1 and experiment 2 in the competitor condition, estimate = −0.059, SE = 0.01, z = −5.28, *p* < 0.001, with lower fixations in experiment 2. However, no significant difference was observed between experiments in the non-competitor condition, estimate = 0.01, SE = 0.01, z = 0.51, *p* = 0.60.

For C2, the analysis for speaker 2 also showed significant effects of experiment and competitor type. The effect of experiment was significant, β = −0.17, SE = 0.05, t = −3.49, *p* = 0.001, indicating that fixation patterns differed between experiments. Competitor type had a significant effect on fixation, β = −0.15, SE = 0.04, t = −3.50, *p* = 0.004, with higher fixations in the non-competitor condition. The interaction between experiment and competitor type was significant, β = 0.13, SE = 0.01, t = 9.76, *p* < 0.001, highlighting that the impact of competitor type on fixation varied between experiments.

Post-hoc contrasts revealed a significant difference in target fixation between experiment 1 and experiment 2 in the competitor condition, estimate = 0.17, SE = 0.05, z = 3.49, *p* < 0.001, with higher fixations in experiment 2. In contrast, no significant difference was found between experiments in the non-competitor condition, estimate = 0.04, SE = 0.05, z-ratio = 0.81, *p* = 0.41.

On the one hand, the eye-tracking analysis revealed the predicted result: participants were more influenced by the competitor objects when they were shared with Confederate 1 in Experiment 2. On the other hand, the unexpected finding was that changes in Confederate 1’s visual field also affected how participants took the perspective of Confederate 2. In particular, the distinct visual field of Confederate 1 served as a reminder to participants that Confederate 2 was unaware of the objects in the blocked compartment. This implies that the comparison of knowledge between the two confederates highlights the importance of recognizing the common ground between the speakers and the participants.

### 3.4 Discussion

In Experiment 2, both confederates sat opposite the participant, acting as speakers and directing the participant in an object-picking task. However, the two confederates had different visual fields: one confederate (C1) shared all four pieces of object information with the participant, whereas the other confederate (C2) did not. Consequently, the two confederates had different levels of shared information. Therefore, this study revealed how participants adjusted their perspective-taking strategies based on their partners’ roles and the information shared.

First, the results confirmed that participants interpreted each confederate’s instructions separately, depending on the information they shared with that particular confederate. Behavioral data showed that although participants tended to choose the shared target in both C1 and C2 trials, the target selection rate was significantly higher in C2 trials than in C1 trials. The eye-tracking data showed similar results: participants’ fixations on the target were significantly higher in C2 trials than in C1 trials. Furthermore, the eye-tracking data revealed more implicit processing by participants. In C1 trials, participants exhibited a competitor effect: fixations on the target were significantly higher in the non-competitor condition compared to the competitor condition. However, this effect was absent in C2 trials. This was because, in C2 trials, a compartment obscured the other candidate object, and participants rarely considered this candidate, which was visible only to themselves. In summary, although confederate 1 and confederate 2 gave identical instructions under the same object arrangement context, participants did not process these instructions consistently. Instead, they engaged in *partner-specific* interpretation based on the shared information with each confederate.

Second, a comparison between two experiments revealed that participants’ perspective-taking with confederate 1 also influenced their interpretation of confederate 2’s instructions. Although behavioral data indicated that participants only exhibited differences in target selection for the competitor condition in C1 trials (and no differences in C2 trials), the eye-tracking data explained the implicit attentional differences underlying these choices. Specifically, in Experiment 2, participants had fewer fixations to the target in C1 trials compared to Experiment 1, but had more fixations to the target in C2 trials. This opposite pattern indicates that an increase in shared information with one confederate heightened participants’ awareness of the lack of shared information with the other. This aligns with previous findings that show similarity with the interlocutor actually leads to less perspective-taking by participants. Since confederate 2 was a less similar interlocutor to the participants, compared to confederate 1, participants took confederate 2’s perspective more effectively than in the baseline situation from Experiment 1, and it is possible that certain expectations are formed even before the conversation begins.

## 4 General discussion

This study investigated how native Mandarin speakers rely on perspective information for referential resolution. Research in this area within a Mandarin-speaking context is still relatively limited, so the first experiment in this study provides a baseline reference for future studies. By using Mandarin bare nouns, the critical noun could refer to the shared object, the blocked object, or both. This study offers insights into how participants may take perspectives to make referential resolution when non-target object referents under indefinite or generic interpretations are acceptable. The results from Experiment 1 showed that participants still used perspective information to interpret bare nouns in a definite manner at a level higher than chance.

Another goal of this study was to establish a conversational scenario to investigate whether participants would rely more on the shared information with each confederate speaker or on the confederates’ role as speakers as a whole. The results supported the former: participants considered the shared information with each speaker individually. In Experiment 2, participants interpreted the instructions from confederate 1 and confederate 2 differently, demonstrating that they distinguished between the perspectives of the two speakers. Additionally, although the results revealed that participants did not view two confederates as a whole, they reinforced the contrast between the speakers’ perspectives. In both experiments, although confederate 2 maintained the same position and visual field, participants’ interpretation of her instructions varied. Specifically, competitor inference was reduced in Experiment 2, particularly in eye-tracking data participants exhibited shorter latencies to move their eyes to the target in the competitor condition, compared with the non-competitor conditions. The results conform to the proposal that people take a stronger perspective of the one who has greater difference from themselves.

Meanwhile, the present study offers some additional issues based on its manipulation. The following will illustrate two issues: visual perspective-taking and multi-party conversations.

### 4.1 Visual perspective-taking

In this study, participants are involved in visual perspective-taking or Level 1 Theory of Mind (ToM). Visual perspective-taking refers to the ability to understand what someone else can or cannot see from their viewpoint. Level 1 ToM is the ability to understand that others might not know what they know. The present study does not concern the difference between the two; for example, the neural mechanisms underlying both visual perspective-taking and theory of mind are overlapping yet distinct (Ogawa and Matsuyama, 2022). However, the present manipulation tested how people infer what others know based on how they can see. That is, the present study focuses on the overlapping parts between these two concepts. Visual perspective-taking or Level 1 ToM ability typically develops early in childhood, before the age of 4, at which point Level 2 Theory of Mind matures (Moll and Tomasello, 2006; Flavell et al., 1981). These abilities are not cognitively demanding, making them feasible for real-time conversational use. They are also closely tied to attention allocation. In a dot perspective-taking experiment, participants viewed images of a figure surrounded by walls with or without dots. When the participants’ perspective aligned with the figure’s, that is, the dots visible to the figure were also visible to the participants, judgments about the figure’s perspective were significantly faster than when perspectives were misaligned, with participants able to see dots behind the figure but the figure unable to see them (Samson et al., 2010). Based on this, Heyes (2014) proposed that such processing might be more about attentional orienting than mentalising. When another’s perspective conflicts with one’s own, cognitive competition and inhibition occur between the two alternatives, similar to the Simon effect.

The results of this study contribute to research on visual perspective, specifically regarding how the consistency between orientation and visible information affects participants’ referential resolution. In Experiment 1, both confederates sat opposite the participant and could not see the blocked objects. The orientation of their seating and the information visible in that direction were consistent. Results showed that participants interpreted both confederates’ instructions similarly, demonstrating partial but significant perspective-taking. However, in Experiment 2, confederate 2 was still seated opposite the participant and could not see the blocked objects, and the alignment between her seating orientation and the visible information remained consistent. Yet, confederate 2’s orientation only matched confederate 1’s seating direction, rather than her visible information. Focusing on participants’ understanding of Confederate 2’s instructions, the results showed a change in participants’ strategies. Specifically, in the second experiment, participants’ understanding of Confederate 2’s speech was less influenced by their privileged ground compared to the first experiment. This change might have been influenced by the difference in what Confederate 1 and Confederate 2 could see, or by the misalignment between Confederate 1’s seating direction and the visible information. These findings suggest that judgments of visual perspective go beyond simply determining whether something is in or out of the visual field and may also involve an attentional adjustment process. During this process, the consistency of different partners, or the alignment between the visual field and the visual direction, may play a role.

### 4.2 Multiparty conversation

One notable difference between this study and previous visual perspective-taking research is the potential impact of the number of conversational partners. In one-to-one conversations with two interlocutors, participants can easily integrate both the seating direction and the visual field of the other. Participants can judge whether the direction their partner faces aligns with the visible information or simply assess whether the direction their partner faces aligns with their own. Such a process is less resource-intensive, and interlocutors can thus more easily exclude their privileged knowledge from the referential domain. However, as the number of collaborators increases, the situation becomes more complex. First, each partner cannot simultaneously occupy the same physical space, implying potential differences in visual perspectives. In this study, although confederates 1 and 2 sat on the same side of the shelf, their views could not be identical, and in Experiment 2, these differences were further amplified due to gaps between the partitions in the shelf. Second, when multiple conversational partners are involved, information can be shared by all, some, or none of the participants. With each additional partner, individuals not only need to consider the common ground between themselves and the new interlocutor, but they may also infer the common ground shared between the other two interlocutors. This process imposes a heavy cognitive load. Additionally, even when each individual’s visual perspective is clear, it is not always evident whose perspective the other partners are taking. In summary, the complexity of perspective-taking in multiparty situations increases exponentially. Therefore, individuals may rely on diverse strategies, in addition to shared knowledge, to navigate multiparty conversations.

The results of this study offer several insights into multiparty conversation, particularly regarding how individuals integrate the perspectives of multiple conversational partners. One strategy is to separately analyse each partner’s perspective. Although this method is cognitively demanding, it remains feasible when the number of participants is small. The findings from Experiment 2 support this strategy, as participants demonstrated differentiated understanding of confederate 1 and 2’s instructions, showing that participants have sufficient cognitive resources to distinguish between the perspectives of different interlocutors.

Another strategy for managing multiple partners in conversation involves taking a unified perspective as the number of participants increases. This could be based on the perspective of one particular individual, such as the less knowledgeable person, or it could involve combining the perspectives of all participants at a certain level. Previous research has shown that when the group size is small, such as when only three individuals are present, speakers tend to communicate according to the perspective of the least knowledgeable person, a strategy known as “Aim Low” (Yoon and Brown-Schmidt, 2014, 2018). As the number of conversational partners increases, individuals may shift to a combined strategy, tailoring their expressions based on the group’s collective knowledge (Yoon and Brown-Schmidt, 2019). Both strategies indicate that addressees either compare or combine the knowledge status of all their conversational partners. The current study’s results support this approach to some extent, as participants compared the perspectives of multiple partners. Specifically, the reduced influence of competitor inference in confederate 2 trials in Experiment 2 suggests that participants engaged in comparison across all partners, leading to a more refined understanding of confederate 2’s perspective.

The finding that the participants compare the perspectives of multiple conversational partners also parallels some previous findings. Studies have shown that speakers in group conversations strive to accommodate multiple perspectives, providing clarifications that might not be necessary in one-on-one conversations. Although group conversation is structurally more complex than one-to-one interactions, research has demonstrated that after interacting with multiple individuals, people’s discourse becomes more comprehensible to others (Fay et al., 2000; Lev-Ari and Sebanz, 2020).

Lastly, but not least, this study offers evidence from the perspective of the addressee in multiparty conversation. Previous studies primarily focus on audience design from the speaker’s perspective, examining how speakers tailor their utterances based on the presence of various listeners. However, it remains unclear whether addressees, as recipients of information, adjust their language comprehension similarly. On the one hand, comprehension and production can follow similar patterns. Participants may not only consider the speaker’s perspective but also take into account the speaker’s strategy, which might involve considering the perspectives of others. For example, if “Rockefeller Centre” is common ground for New Yorkers A and B but unfamiliar to C, B might interpret A’s reference to a “building with flags of various nations” differently depending on whether C is present. If C is absent, B might think A is referring to another building. If C is present, B might infer that A is indeed referring to Rockefeller Centre. In this scenario, B’s comprehension adapts not only based on C’s perspective but also on A’s communicative strategy. On the other hand, strategies for processing common ground during production and comprehension may differ. While the speaker directs discourse to multiple individuals, the addressee only processes information from one person at each turn. In this sense, perspective-taking for addressees in multiparty settings may resemble that in one-on-one conversations. The results of this study seem to support the latter, as participants adjusted their perspective-taking strategies based on confederate 1 and 2’s knowledge separately. However, it may be due to the small group size. Future studies can explore how addressees adjust their strategy to accommodate a larger amount of perspectives.

## 5 Conclusion

The present study, through two experiments, examined how participants utilised the perspective information of two speakers to perform referential resolution of nouns. First, this research provides evidence from a Mandarin context, particularly addressing the role of bare nouns, which do not require determiners in Mandarin. The results show that participants largely considered perspective information during referential resolution. Second, the study revealed that participants, as addressees, not only relied on distinct perspectives but also made comparisons between the speakers’ perspectives. This finding aligns with previous production-based studies and offers further evidence for perspective-taking in multiparty conversations from a comprehension standpoint. Third, this research focused primarily on visual perspective and small conversational groups. Future studies could expand on this by exploring more complex forms of perspective information and larger conversational groups, which may involve even more dynamic processes.

## Data Availability

The datasets presented in this study can be found in online repositories. The names of the repository/repositories and accession number(s) can be found a: https://osf.io/y3xcg/?view_only=fd8941f5f81f49299a5ec9cbc9a18def.
